# Remodeling of the tumor microenvironment using an engineered oncolytic vaccinia virus improves PD-L1 inhibition outcomes

**DOI:** 10.1042/BSR20204186

**Published:** 2021-06-10

**Authors:** Jiaying Lou, Jialin Dong, Ruijun Xu, Hui Zeng, Lijuan Fang, Yi Wu, Yang Liu, Shibing Wang

**Affiliations:** 1 Department of Laboratory Medicine, Hangzhou Ninth People’s Hospital, Hangzhou, China; 2 Department of Hematology, Zhejiang Provincial People’s Hospital, People’s Hospital of Hangzhou Medical College, Hangzhou, China; 3 Department of Ultrasonography, Zhejiang Provincial People’s Hospital, People’s Hospital of Hangzhou Medical College, Hangzhou, China; 4 Molecular Diagnosis Laboratory, Zhejiang Provincial People’s Hospital, People’s Hospital of Hangzhou Medical College, Hangzhou, China; 5 The Key Laboratory of Tumor Molecular Diagnosis and Individualized Medicine of Zhejiang Province, Zhejiang Provincial People’s Hospital, People’s Hospital of Hangzhou Medical College, Hangzhou, China

**Keywords:** lymphoma, MnSOD, Oncolytic vaccinia virus, PD-L1, Tumor microenvironment

## Abstract

Immune checkpoint inhibitor (ICI) immunotherapies have vastly improved therapeutic outcomes for patients with certain cancer types, but these responses only manifest in a small percentage of all cancer patients. The goal of the present study was to improve checkpoint therapy efficacy by utilizing an engineered vaccinia virus to improve the trafficking of lymphocytes to the tumor, given that such lymphocyte trafficking is positively correlated with patient checkpoint inhibitor response rates. We developed an oncolytic vaccinia virus (OVV) platform expressing manganese superoxide dismutase (MnSOD) for use as both a monotherapy and together with anti-PD-L1. Intratumoral OVV-MnSOD injection in immunocompetent mice resulted in inflammation within poorly immunogenic tumors, thereby facilitating marked tumor regression. OVV-MnSOD administration together with anti-PD-L1 further improved antitumor therapy outcomes in models in which these monotherapy approaches were ineffective. Overall, our results emphasize the value of further studying these therapeutic approaches in patients with minimally or non-inflammatory tumors.

## Introduction

The development of immune checkpoint inhibitor (ICI) immunotherapies including anti-programmed cell death-1 (PD-1)/PD-L1 and anti-cytotoxic T lymphocyte antigen 4 (CTLA4) have significantly improved survival and clinical outcomes in a subset of patients with certain tumor types [1–3]. In a study of lymphoma patients, ICI treatment exhibited synergistic activity with other antitumor therapies relative to monotherapeutic interventions, but these treatments were associated with systemic toxicities associated with the robust activation of the immune system [4]. Further research is thus required to optimize the clinical efficacy of these therapeutic approaches while minimizing toxicity via utilizing different ICIs, agonistic drugs, cytokines, and combinations thereof [5,6].

The local administration of immunotherapeutic agents represents an ideal approach to minimizing off-target systemic toxicity. The use of tumor-specific oncolytic viruses (OVs) capable of replicating in the tumor microenvironment (TME) and killing tumor cells to release tumor-specific antigens has the potential to improve cancer treatment outcomes [7,8]. Such virus-induced tumor cell lysis can also activate innate immune receptors owing to the concomitant release of damage-associated molecular patterns, thereby bolstering antitumor immune responses [9]. Tumor-selective OVs can also be leveraged as vectors to deliver therapeutic genes to the TME, and several such viruses have been tested in this context. For example, talimogene laherparepvec (T-VEC) [10,11], which is a herpes simplex virus strain that was engineered to encode the immune adjuvant granulocyte-macrophage colony-stimulating factor (GM-CSF), was approved as a first-in-class OV in U.S.A. in 2015 to treat metastatic melanoma. Phase III trials have also been conducted to assess the efficacy of the GM-CSF-encoding Pexastimogene devacirepvec (Pexa-Vec, JX-594) in advanced hepatocellular carcinoma patients [12,13]. Whether the encoding of these immunomodulatory genes improves overall patient clinical outcomes, however, remains uncertain, as these genes can promote local inflammation that suppresses the oncolytic activity of the viral vectors and further suppresses the expression of these transgenes [14]. In addition to directly lysing tumor cells, OVs have been shown to effectively activate immune cells and promote their infiltration into tumors, circumventing immunosuppression in the microenvironment. Considering the immunomodulatory properties of OVs, strategies combining virotherapy with other immunotherapies, such as ICIs, have been proposed [15–18]. OV-based immunotherapy combined strategy can elicit tumor killing effect via multiple targets and mechanisms, which may be expected to improve the situation [19].

We have previously demonstrated the ability of oncolytic vaccinia virus (OVV), which is a promising immune-oncolytic therapy strategy [20], to induce T cell and NK cell infiltration and to reduce myeloid-derived suppressor cell (MDSC) numbers in a model of subcutaneous lymphoma [21]. We also generated the novel tumor-homing E1B55K gene-deleted ZD55-MnSOD oncolytic adenovirus encoding the manganese superoxide dismutase (*MnSOD*) gene, and we found that this virus was able to promote robust tumor cell death *in vitro* and *in vivo* in models of ovarian and colorectal cancer [22]. Several studies have shown that MnSOD^−/+^ mice are prone to spontaneous lymphoma development and MnSOD is crucial for proper thymocyte differentiation, homeostatic survival of peripheral T cells as well as for T cell-mediated immune responses [23–25]. So we presumed that overexpression of MnSOD not only suppresses tumor cell growth, but also promotes stronger antitumor immune response. Herein, we sought to explore the intratumoral changes in immune status when utilizing an MnSOD-expressing OVV platform either alone or in combination with anti-PD-L1. Through these experiments, we found that OVV-MnSOD markedly enhanced intratumoral inflammation, improved systemic antitumor efficacy, and increased lymphoma sensitivity to PD-L1 blockade. Together, our results highlight novel approaches to overcoming ICI resistance in tumors, providing a foundation for efforts to expand these results to human clinical trials.

## Materials and methods

### OVV-MnSOD synthesis

OVV and OVV-MnSOD vectors were prepared via gene recombination [26,27]. Briefly, the full MnSOD gene sequence was then inserted into the pCB plasmid. The resultant sequenced pCB-MnSOD or control pCB vectors were then transfected into HEK293A cells that had already undergone WT vaccinia virus infection. In brief, HEK293 cells were infected with WT vaccinia virus at a multiplicity of infection (MOI) of 1 for 2 h and then transfected with the corresponding shuttle plasmid. The cell extraction solution was used to infect the HEK293 cells in the presence of 25 mg/ml mycophenolic acid (MPA; Cat# A600640, Sangon Biotech, Shanghai, China), 250 mg/ml xanthine (Cat# A601197, Sangon Biotech), and 15 mg/ml hypoxanthine (Cat# A500336, Sangon Biotech). After three cycles of screening, EGFP-positive plaques were isolated, resuspended, and further HEK293 cells were infected for two cycles of plaque purification. After completing the first and second rounds of plaque purification, the following primers were used to amplify the target gene and the viral thymidine kinase (*TK*) gene to identify whether the recombinant virus was adulterated with the parental vaccinia virus. Primer of target gene: 5′-CTCCCCGACCTGCCCTACGACT-3′, 5′-TGCAAGCCATGTATCTTTCAGTTAC-3′; primer of TK: 5′-tgtgaagacgataaattaatgatc-3′, 5′-gtttgccatacgctcacag-3′. Recombinant vaccinia virus successfully screened by plaque purification was further expanded by Hela-S3 cells in six-well plates, cell culture dishes, and cell culture spinner flasks.

### Quantitative RT-PCR

TRIzol (#15596-026; Invitrogen) was utilized to extract total RNA from infected cells, after which PrimeScript RT Master Mix (#DRR036A, TaKaRa, Shiga, Japan) was used to prepare cDNA. Next, FastStart Universal SYBR Green Master Mix (#04913914001; Roche) was employed to conduct quantitative RT-PCR (qPCR) with a Real-Time PCR System (Applied Biosystems, CA, U.S.A.). The comparative *C*_t_ method was used to evaluate relative gene expression, with GAPDH for normalization.

### Western blot

Cells were lysed using RIPA buffer and BCA assay was then conducted to measure protein levels in these samples based on the provided directions. Equal protein amounts were separated via 10–15% SDS/PAGE and transferred on to PDF membranes that were probed with anti-MnSOD (Abcam, ab68155), anti-Caspase-3 (Abcam, ab3251), or anti-GAPDH (Abcam, ab9485). Blots were then probed with HRP-linked secondary antibodies for 1 h (1:4000; HuaAn Biotechnology Co. Ltd). The ImageJ software was then employed to measure protein band density.

### CCK8 assay

Murine lymphoma A20 and EL4 cells were added to 960-well-plates (10000 cells/well) and were infected with OVV or OVV-MnSOD for 48 h, after which 10 μl of CCK8 reagent was added per well for 4 h at 37°C. Absorbance at 450 nm was then assessed with a microplate reader to assess the cytolytic activity of these viral preparations. PBS was used as a negative control.

### Animal experiments

The Animal Ethics Committee of Zhejiang Provincial People’s Hospital approved all animal studies (A20190029). C57BL/6 mice were purchased from Zhejiang Chinese Medical University, Hangzhou, China) and housed in a specific pathogen-free facility with free food and water access in Animal Laboratory of Zhejiang Provincial People’s Hospital. Digital calipers were used to measure subcutaneous tumor diameter, with tumor volume being defined as: volume = length × width^2^ × 0.5. The body weight and tumor sizes of all mice were regularly monitored, and mice were killed by carbon dioxide suffocation if they exhibited acute weight loss or tumors ≥ 3000 mm^3^ in size. When tumors were no longer palpable, mice were considered to have achieved a complete response (CR).

A20 and EL4 cells (5 × 10^6^ or 2 × 10^6^ cells, respectively) were subcutaneously injected into the right flank of model mice. After tumors were ∼50 mm^3^ in size, 50 μl of PBS, OVV, or OVV-MnSOD were injected into the tumor every other day (three doses in total). Combination therapy efficacy was assessed by also intraperitoneally injecting these mice with 100 μg of anti-PD-L1 (clone RMP1-14, Bio X Cell) beginning on the second day of initial viral treatment every 3 days for three times.

### Flow cytometry

A mouse Tumor Dissociation Kit (Miltenyi Biotec) was used to prepare cells, which were then stained with the following antibodies: mouse CD3 V450, mouse CD8a V500, mouse CD11b APC-Cy7, mouse CD25 PE–Cy7, mouse CD80 PE, mouse I-A/I-E PerCP–Cy5.5, and mouse Ly-6G FITC (BD Biosciences); mouse B220 FITC, mouse CD3 APC-Cy7, mouse CD4 FITC, mouse CD11c PE, mouse CD44 PE-Cy7, mouse CD45 Alexa Fluor 647, mouse CD45 APC. Then cells were analyzed with an FACS Caliber cytometer (BD). Data analyses were performed with FlowJo software (Treestar, U.S.A.).

### Statistical analysis

SPSS 17.0 (IBM Corp., NY, U.S.A.) and GraphPad Prism (GraphPad Software, Inc.) were used for statistical testing. Continuous data are given as means ± standard deviation (SD), and were compared through unpaired two-tailed Student’s *t* tests or two-way analyses of variance (ANOVAs) with Tukey’s multiple comparisons test. Kaplan–Meier curves were used to assess survival outcomes. *P*<0.05 was the significance threshold.

## Results

### OVV-*MnSOD* characterization

**Figure 1:**
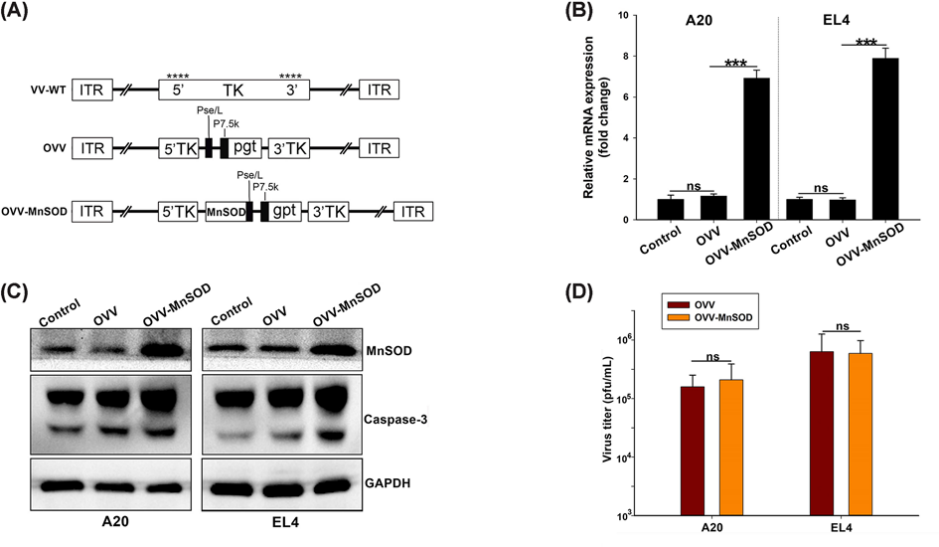
Construction and characterization of OVV-*MnSOD* (**A**) Linear schematic of OVV-MnSOD structure. All viruses were constructed through homologous recombination between pCB-transgene and wildtype vaccinia virus (VV) in HEK293A cells. The *MnSOD* expression cassette was introduced into the TK region of vaccinia virus. Pse/L, synthetic early/late promoter. P7.5, vaccinia virus early-late promoter. gpt, mycophenolic acid resistance gene. 5′TK and 3′TK, viral flanking sequences of the TK gene. ITR, inverted terminal repeat. ****, sites of anticipated homologous recombination. (**B,C**) The expression of *MnSOD*. Cells on six-well plates were infected with different viruses at an MOI of 2. After 48 h post-infection, total cellular RNA and protein lysates were extracted to elevate the *MnSOD* expression using real-time PCR and Western blot. (**B**) GAPDH served as an internal control. The data are presented as the mean ± SD of three separate experiments (*** represents *P*<0.001, one-way ANOVA and multiple comparisons). (**C**) Western blot analysis of the apoptosis-related protein caspase in A20 and EL4 cells. β-actin served as a loading control. (**D**) A20 and EL4 cells were infected with MOI: 1 of OVV-MnSOD or OVV, respectively. After an additional 48 h, medium and cells were harvested. The collected supernatant was tested for virus production by standard TCID_50_ assay on 293A cells. Progeny viruses from MOI: 1 of virus were calculated. Data are presented as mean ± SD and the representative of three separate experiments.

We employed a homologous recombination approach to prepare OVV-MnSOD as detailed previously [28], and as shown in Figure 1A. Successful exogenous expression of *MnSOD* in these viral particles was confirmed via qPCR. OVV and OVV-MnSOD were then used to infect A20 and EL4 lymphoma cells (MOI = 2) for 24 h, after which significant *MnSOD* expression was detectable in cells infected with OVV-MnSOD but not in cells infected with OVV or treated with PBS (Figure 1B). Western blotting yielded comparable results regarding MnSOD protein levels in these cells, confirming that we had successfully prepared an OVV strain capable of overexpressing MnSOD in target cells. Subsequent Western blotting also revealed that OVV-MnSOD infection significantly induced caspase-3 cleavage in both the cell lines at 48 h post-infection, consistent with the apoptotic death of these target cells (Figure 1C).

As an additional control, we evaluated the impact of MnSOD expression on OVV viral replication in these two lymphoma cell lines. This analysis revealed no significant difference in viral yield when comparing the OVV and OVV-MnSOD viruses, suggesting that this transgene had no adverse impact on such replication (Figure 1D). As such, the ability of these OVV preparations to selectively replicate within tumor cells is unaffected by the deletion of TK or the insertion of the MnSOD transgene.

### *In vitro* antitumor activity of OVV-*MnSOD*

**Figure 2:**
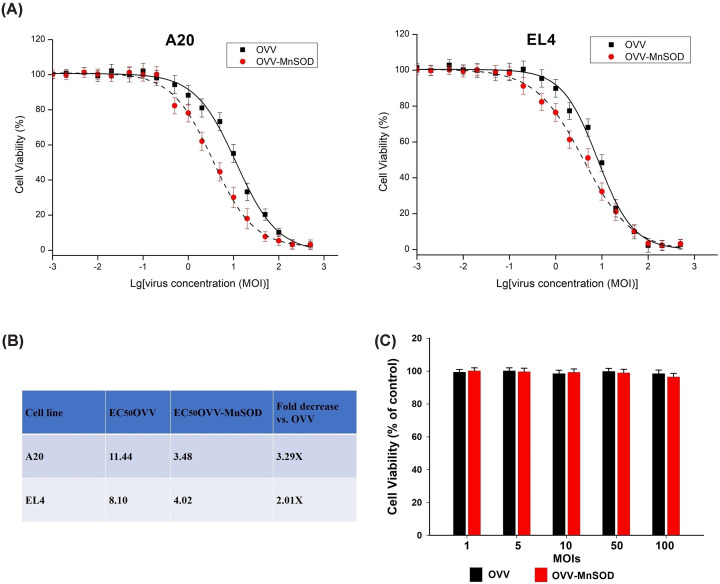
*In vitro* antitumor activity of OVV-MnSOD Cells were seeded on 96-well plates and infected with OVV or OVV-*MnSOD* at the indicated MOIs. (**A**) Cytotoxicity and (**B**) IC_50_ of oVV and oVV-Smac in two lymphoma cell lines was determined by CCK8 cell proliferation assay at 48 h post-infection and expressed as the percentage of absorbance of treated cells with respect to that of mock-treated samples. (**C**) PBMCs are used to verify the security of the OVV-MnSOD according to (A). The results are presented as mean ± SD of three separate experiments.

A 3-(4,5-dimethylthiazol-2-yl)-2,5-diphenyltetrazolium bromide (MTT) assay was next conducted using A20 and EL4 cells following a 48-h infection with OVV-MnSOD in order to gauge the cytotoxic activity of this virus. OVV-MnSOD exhibited superior inhibition of lymphoma cell proliferation relative to OVV in a dose-dependent manner in this assay (Figure 2A,B), whereas neither virus was able to suppress peripheral blood mononuclear cell (PBMC) replication (Figure 2C). Overall, these findings indicated that OVV-MnSOD can selectively and specifically suppress the *in vitro* growth of tumor cells.

### OVV-MnSOD promotes lymphocyte infiltration into tumors

**Figure 3:**
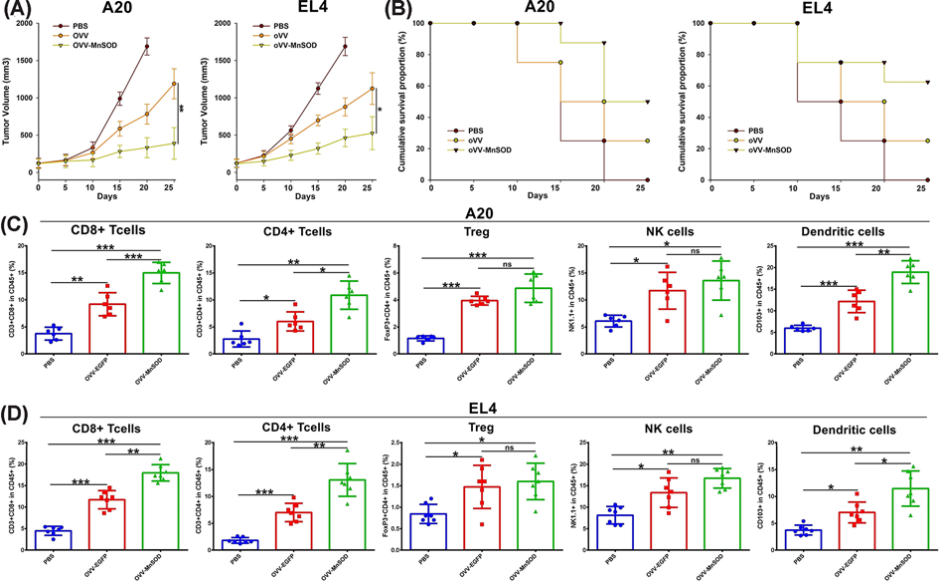
OVV-MnSOD shows antitumor responses and increases in tumor-infiltrating lymphocytes (**A**) Mice were subcutaneously inoculated with A20 or EL4 cells. When tumors reached approx. 50 mm^3^, tumors were directly injected with PBS, OVV or OVV-MnSOD (2 × 10^7^ pfu) every other day, for a total of three injections. Tumor growth in mice treated with PBS, OVV, or OVV-MnSOD is shown (*n*=12 for A20, *n*=13 for EL4 per group). *P<0.05, *P<0.05, and ***P<0.001 by unpaired *t* test. (**B**) Kaplan–Meier survival curves. (**C,D**) Seven days after the last treatment, tumors were collected and analyzed by flow cytometry to calculate the percentages of TILs in tumor cells. Intratumoral CD8^+^ T cells, CD4^+^ T cells, Treg, NK cells, and dendritic cells in A20 (C) and EL4 (D) tumor models (D). *n*=6 for A20 and *n*=7 for EL4. **P<0.01 and ***P<0.001 by Mann–Whitney U test. ns, not significant. Mean ± SD is shown. Abbreviation: TIL, tumor-infiltrating lymphocyte.

In order to establish the ability of our virotherapy approach to promote remodeling within the TME, we next intratumorally administered OVV or OVV-MnSOD into immunocompetent mice bearing A20 or EL4 tumors (Supplementary Figure S1). In the former model, OVV-MnSOD resulted in an 84.9% inhibition of tumor growth relative to 53.8% for OVV, while in the latter tumor model these percentages were 77.8 and 52.5%, respectively (Figure 3A). Animals were killed when tumors were over 2000 mm^3^ in size or mice appeared moribund. All mice administered an intratumoral PBS injection were killed within 20 days of treatment, while 75% of A20 model mice and 62.5% of EL4 model mice treated with OVV-MnSOD survived (Figure 3B).

To establish the impact of OVV-MnSOD on tumor-infiltrating lymphocyte (TIL) populations, we conducted flow cytometry on tumor samples collected on day 7 following the third treatment of these mice. Tumor-derived single-cell suspensions were stained with antibodies specific for CD45, CD3, CD4, CD8, FoxP3, NK1.1, and CD103. This analysis revealed that OVV-MnSOD administration was associated with a significant increase in the frequency of CD8^+^ T cells, CD4^+^ T cells, Treg, NK cells, and dendritic cells (DCs) within EL4 and A20 tumors relative to those treated with PBS, and to increase the frequency of CD8^+^ T cells, CD4^+^ T cells, and DCs in both of these tumor types relative to OVV treatment (Figure 3C,D).

### OVV-MnSOD enhances tumor sensitivity to anti-PD-L1 treatment

**Figure 4:**
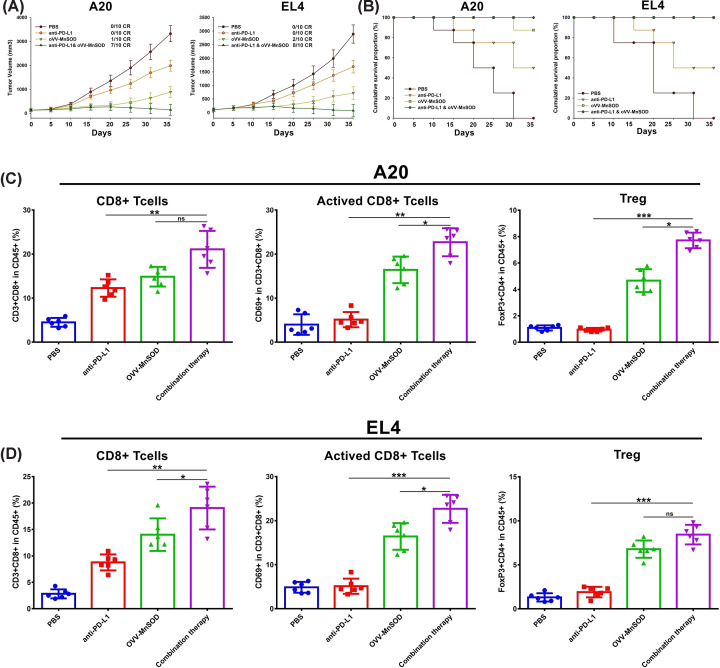
Intratumoral administration of OVV-MnSOD sensitizes tumors to anti-PD-L1 antibodies (**A**) Mouse lymphoma cells A20 or EL4 were subcutaneously implanted into C57/BL6 mouse before treatments. Tumors were treated, beginning when tumors reached 50 mm^3^, with vehicle solution or 2 × 10^7^ pfu of OVV-MnSOD or OVV, for a total of three times. From the day of the first injection of virus, 100 μg of anti-PD-L1 antibody was intraperitoneally administered every 3 days during the experiment. (**B**) Over the course of treatment with either OVV-MnSOD, OVV or PBS, tumor volumes were measured. (*n*=10 for A20 and EL4 per group). (B) Kaplan–Meier survival curves. (**C,D**) In a separate study, 10 days after the last viral treatment, infiltrating CD8^+^ T cells, activated CD8^+^ T cells, and Treg in injected tumors were analyzed by flow cytometry (*n*=5–6). *P<0.05, **P<0.01, and ***P<0.001 by Mann–Whitney U test. Mean ± SD is shown.

We next speculated that the increases in TIL levels within tumors following OVV-MnSOD injection would enhance the susceptibility of these tumors to ICI treatment. To test this, we treated mice bearing these lymphoma model tumors with anti-PD-L1 with or without prior intratumoral OVV-MnSOD treatment (Supplementary Figure S2). While anti-PD-L1 monotherapeutic treatment exhibited limited efficacy and did not facilitate CR in any of the treated mice, combination OVV-MnSOD + anti-PD-L1 treatment was associated with CR in the majority of treated mice (7/10 CR in the A20 model, 8/10 in the EL4 model) (Figure 4A). Importantly, 100% of mice in both of these combination treatment groups survived the study period (Figure 4B). Intratumoral injection of OVV-MnSOD prior to PD-L1 blockade increased CD8^+^ T cells, activated CD8^+^ T cells, and Tregs in A20 (Figure 4C) and EL4 (Figure 4D) tumors. This suggests that locally injecting OVV-MnSOD can increase tumor sensitivity to ICI treatment. No murine weight loss was detected over the course of the study period (data not shown).

## Discussion

A range of immunotherapies has been tested to treat lymphoma patients. ICI-based treatments including anti-PD-1/PD-L1, anti-CTLA4, and combinations thereof are increasingly common standards of care for certain forms of cancer [29]. However, these immune checkpoint blockage approaches are only efficacious in a limited number of patients owing to the potential for systemic toxicity and the role of multiple different immune signaling pathways within the TME [30]. It is thus essential that novel approaches to safely and effectively improving tumor patient clinical response rates be developed. In the present study, we found that intratumoral OVV-MnSOD administration both altered the local and the systemic immune status in mice bearing lymphoma model tumors refractory to anti-PD-L1 treatment. Importantly, this virotherapy approach was sufficient to sensitize mice to ICI treatment without inducing significant toxicity.

Increased mitochondrial reactive oxygen species (ROS) production is a common hallmark of tumor cells. After being produced, ROS can suppress T-cell activation and proliferation within the TME such that high levels of ROS can impair the development of antitumor immune responses in cancer patients, whereas low levels of ROS can enhance T cell receptor-induced signal transduction [31,32]. MnSOD is a mitochondrial antioxidant protein found in *Escherichia coli* and encoded by nuclear genes on chromosome 6q21. Reductions in MnSOD activity are frequently observed in tumor tissues and cell lines from humans, and it has been shown to function as a tumor suppressor protein such that MnSOD overexpression can suppress the growth of a range of tumor types [33,34].

There are multiple limitations to the present study that should be considered when evaluating this therapeutic approach. For one, while we were able to demonstrate OVV-MnSOD-induced changed in the local tumor microenvironmental immune status based on analyses of particular cell subsets; further work is necessary to fully understand the functional roles of cells such as antigen-presenting cells, stromal cells, and Tregs within tumors in the context of this virotherapy approach. In particular, more work is needed to understand the link between OVV therapy and adaptive antitumor immunity through gene expression and T cell repertoire analyses [14]. In addition, more research is required to establish the tumor types that are most likely to be amenable to virotherapy treatment by identifying key therapeutic biomarkers. While we initiated anti-PD-L1 treatment on the first day of OVV-MnSOD administration in light of prior reports suggesting that ICIs can suppress vaccinia virus replication, additional dosing optimization is required to improve the clinical outcomes associated with this therapeutic strategy. Clinical trials evaluating the combination of ICIs with OVs, such as T-VEC followed by pembrolizumab, will provide key insights regarding the promise of such combination virotherapy.

Together, our results suggest that the intratumoral administration of OVV-MnSOD can enhance antitumor immunity and increase tumor immunogenicity and amenability to checkpoint blockade therapy. This OVV-MnSOD preparation may offer value as both a monotherapy and together with different immunotherapies when used to treat diverse tumor types.

## Supplementary Material

Supplementary Figures S1-S2

## Data Availability

Some or all data generated or used during the study are available from the corresponding authors on request.

## Abbreviations

CR: complete response
CTLA4: cytotoxic T lymphocyte antigen 4
GM-CSF: granulocyte-macrophage colony-stimulating factor
ICI: immune checkpoint inhibitor
MnSOD: manganese superoxide dismutase
MOI: multiplicity of infection
OV: oncolytic virus
OVV: oncolytic vaccinia virus
PD-1: programmed cell death-1
PD-L1: programmed cell death-Ligand 1
qPCR: quantitative RT-PCR
ROS: reactive oxygen species
TIL: tumor-infiltrating lymphocyte
TK: thymidine kinase
TME: tumor microenvironment
Treg: regulatory T cells
T-VEC: talimogene laherparepvec

## References

[1] Goodman A. et al. (2017) PD-1-PD-L1 immune-checkpoint blockade in B-cell lymphomas. Nat. Rev. Clin. Oncol. 14, 203–220 10.1038/nrclinonc.2016.168 27805626

[2] Jelinek T. et al. (2018) Update on PD-1/PD-L1 inhibitors in multiple myeloma. Front. Immunol. 9, 2431 10.3389/fimmu.2018.02431 30505301 PMC6250817

[3] Jelinek T. et al. (2017) PD-1/PD-L1 inhibitors in haematological malignancies: update 2017. Immunology 152, 357–371 10.1111/imm.12788 28685821 PMC5629439

[4] Xu-Monette Z.Y. et al. (2018) PD-1 expression and clinical PD-1 blockade in B-cell lymphomas. Blood 131, 68–83 10.1182/blood-2017-07-740993 29118007 PMC5755041

[5] Schmidt C. (2017) The benefits of immunotherapy combinations. Nature 552, S67–S69 10.1038/d41586-017-08702-7 29293245

[6] Chen D.S. and Mellman I. (2017) Elements of cancer immunity and the cancer-immune set point. Nature 541, 321–330 10.1038/nature21349 28102259

[7] Kaufman H.L. et al. (2015) Oncolytic viruses: a new class of immunotherapy drugs. Nat. Rev. Drug Discov. 14, 642–662 10.1038/nrd4663 26323545 PMC7097180

[8] Russell S.J. and Barber G.N. (2018) Oncolytic viruses as antigen-agnostic cancer vaccines. Cancer Cell 33, 599–605 10.1016/j.ccell.2018.03.011 29634947 PMC5918693

[9] Lawler S.E. et al. (2017) Oncolytic viruses in cancer treatment: a review. JAMA Oncol. 3, 841–849 10.1001/jamaoncol.2016.2064 27441411

[10] Killock D. (2015) Skin cancer: T-VEC oncolytic viral therapy shows promise in melanoma. Nat. Rev. Clin. Oncol. 12, 438 10.1038/nrclinonc.2015.106 26077044

[11] Blake Z. et al. (2018) Complete intracranial response to talimogene laherparepvec (T-Vec), pembrolizumab and whole brain radiotherapy in a patient with melanoma brain metastases refractory to dual checkpoint-inhibition. J. Immunother. Cancer 6, 25 10.1186/s40425-018-0338-6 29622046 PMC5887256

[12] Park S.H. et al. (2015) Phase 1b trial of biweekly intravenous Pexa-Vec (JX-594), an oncolytic and immunotherapeutic vaccinia virus in colorectal cancer. Mol. Ther. 23, 1532–1540 10.1038/mt.2015.109 26073886 PMC4817877

[13] Heo J. et al. (2011) Sequential therapy with JX-594, a targeted oncolytic poxvirus, followed by sorafenib in hepatocellular carcinoma: preclinical and clinical demonstration of combination efficacy. Mol. Ther. 19, 1170–1179 10.1038/mt.2011.39 21427706 PMC3129795

[14] Lun X.Q. et al. (2009) Efficacy of systemically administered oncolytic vaccinia virotherapy for malignant gliomas is enhanced by combination therapy with rapamycin or cyclophosphamide. Clin. Cancer Res. 15, 2777–2788 10.1158/1078-0432.CCR-08-2342 19351762

[15] Engeland C.E. et al. (2014) CTLA-4 and PD-L1 checkpoint blockade enhances oncolytic measles virus therapy. Mol. Ther. 22, 1949–1959 10.1038/mt.2014.160 25156126 PMC4429737

[16] Liu Z. et al. (2017) Rational combination of oncolytic vaccinia virus and PD-L1 blockade works synergistically to enhance therapeutic efficacy. Nat. Commun. 8, 14754 10.1038/ncomms14754 28345650 PMC5378974

[17] Cervera-Carrascon V. et al. (2020) Tumor microenvironment remodeling by an engineered oncolytic adenovirus results in improved outcome from PD-L1 inhibition. Oncoimmunology 9, 1761229 10.1080/2162402X.2020.1761229 32923123 PMC7458667

[18] Ribas A. et al. (2018) Oncolytic virotherapy promotes intratumoral T cell infiltration and improves anti-PD-1 immunotherapy. Cell 174, 1031–1032 10.1016/j.cell.2018.07.035 30096300

[19] Twumasi-Boateng K. et al. (2018) Oncolytic viruses as engineering platforms for combination immunotherapy. Nat. Rev. Cancer 18, 419–432 10.1038/s41568-018-0009-4 29695749

[20] Guo Z.S. et al. (2019) Vaccinia virus-mediated cancer immunotherapy: cancer vaccines and oncolytics. J. Immunother. Cancer 7, 6 10.1186/s40425-018-0495-7 30626434 PMC6325819

[21] Wang P. et al. (2020) Embelin promotes oncolytic Vaccinia virus-mediated antitumor immunity through disruption of IL-6/STAT3 signaling in lymphoma. Onco Targets Ther. 13, 1421–1429 10.2147/OTT.S209312 32110041 PMC7034962

[22] Wang S. et al. (2016) Synergistic suppression effect on tumor growth of ovarian cancer by combining cisplatin with a manganese superoxide dismutase-armed oncolytic adenovirus. Onco Targets Ther. 9, 6381–6388 10.2147/OTT.S113014 27799786 PMC5074737

[23] Van Remmen H. et al. (2003) Life-long reduction in MnSOD activity results in increased DNA damage and higher incidence of cancer but does not accelerate aging. Physiol. Genomics 16, 29–37 10.1152/physiolgenomics.00122.2003 14679299

[24] Ji G. et al. (2012) Genetic variants in antioxidant genes are associated with sperm DNA damage and risk of male infertility in a Chinese population. Free Radic. Biol. Med. 52, 775–780 10.1016/j.freeradbiomed.2011.11.032 22206979

[25] Kaminski M.M. et al. (2012) Manganese superoxide dismutase: a regulator of T cell activation-induced oxidative signaling and cell death. Biochim. Biophys. Acta 1823, 1041–1052 10.1016/j.bbamcr.2012.03.003 22429591

[26] Dana H. (2017) Genetically engineered Vaccinia viruses as agents for cancer treatment, imaging, and transgene delivery. Front. Oncol. 7, 96– 10.3389/fonc.2017.00096 28589082 PMC5440573

[27] Deng L. et al. (2017) Oncolytic efficacy of thymidine kinase-deleted vaccinia virus strain Guang9. Oncotarget 8, 40533–40543 10.18632/oncotarget.17125 28465492 PMC5522336

[28] Lei W. et al. (2016) Combined expression of miR-34a and Smac mediated by oncolytic vaccinia virus synergistically promote anti-tumor effects in multiple myeloma. Sci. Rep. 6, 32174 10.1038/srep32174 27552933 PMC5001249

[29] Davids M.S. et al. (2016) Ipilimumab for patients with relapse after allogeneic transplantation. N. Engl. J. Med. 375, 143–153 10.1056/NEJMoa1601202 27410923 PMC5149459

[30] Sharma P. and Allison J.P. (2015) The future of immune checkpoint therapy. Science 348, 56–61 10.1126/science.aaa8172 25838373

[31] Xiang H. et al. (2020) Cancer-associated fibroblasts promote immunosuppression by inducing ROS-generating monocytic MDSCs in lung squamous cell carcinoma. Cancer Immunol. Res. 8, 436–450 10.1158/2326-6066.CIR-19-0507 32075803

[32] Weinberg F. et al. (2019) Reactive oxygen species in the tumor microenvironment: an overview. Cancers (Basel) 11, 1–20 10.3390/cancers11081191 PMC6721577 31426364

[33] Oberley L.W. (2005) Mechanism of the tumor suppressive effect of MnSOD overexpression. Biomed. Pharmacother. 59, 143–148 10.1016/j.biopha.2005.03.006 15862707

[34] Zhang R. et al. (2016) Enhanced antitumor effect of combining TRAIL and MnSOD mediated by CEA-controlled oncolytic adenovirus in lung cancer. Cancer Gene Ther. 23, 168–177 10.1038/cgt.2016.11 27080225

