# Correction: Remodeling of the tumor microenvironment using an engineered oncolytic vaccinia virus improves PD-L1 inhibition outcomes

**DOI:** 10.1042/BSR-2020-4186_COR

**Published:** 2022-12-09

**Keywords:** lymphoma, MnSOD, Oncolytic vaccinia virus, PD-L1, Tumor microenvironment

The authors of the original article “Remodeling of the tumor microenvironment using an engineered oncolytic vaccinia virus improves PD-L1 inhibition outcomes” (*Biosci Rep*. 2021 **41**(6): BSR20204186. https://doi.org/10.1042/BSR20204186) would like to correct Figure 1C. Due to the unstandardised naming and storage conventions of western blot images and an error in the authors' figure creation, an incorrect image was selected. The authors declare that these corrections do not change the results or conclusions of their paper, and express their sincere apologies for any inconvenience that this error has caused to the readers. The corrected version of Figure 1 is presented here.

**Figure 1:**
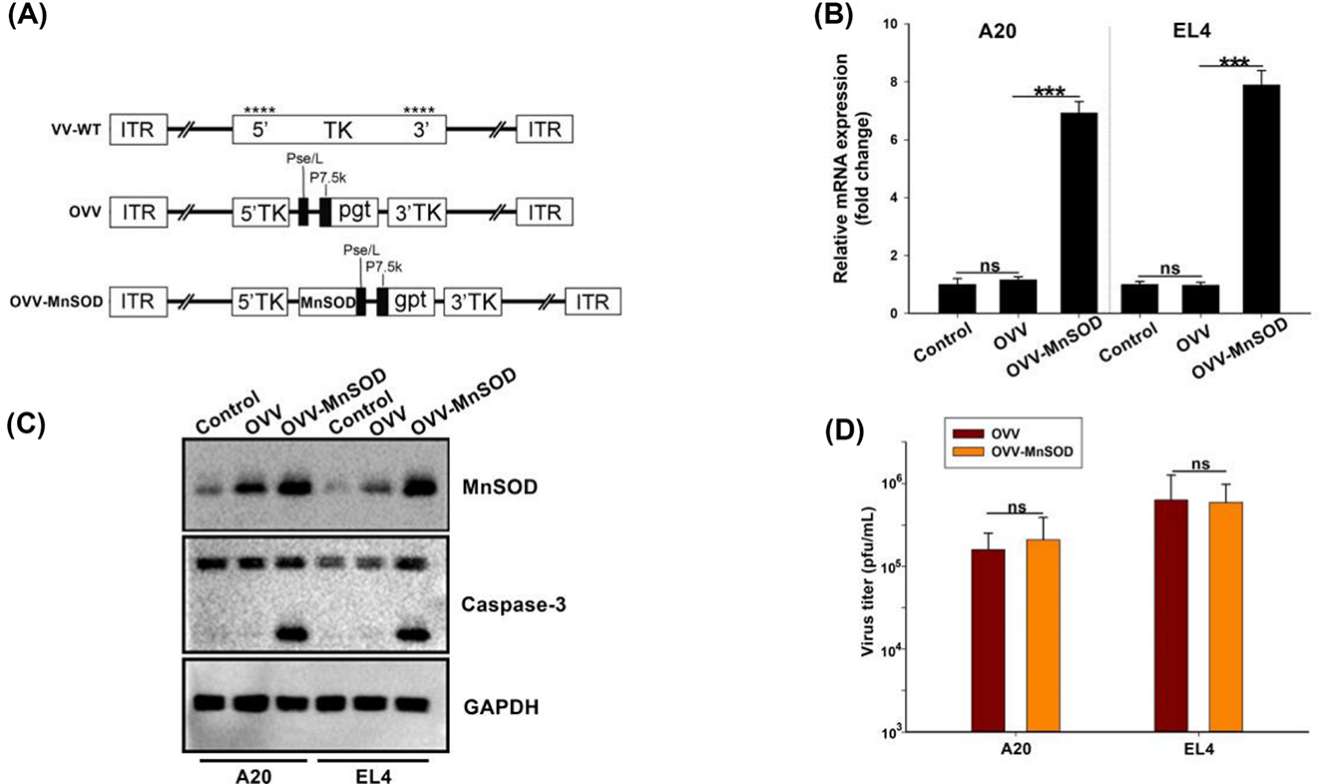
Construction and characterization of OVV-*MnSOD* (**A**) Linear schematic of OVV-MnSOD structure. All viruses were constructed through homologous recombination between pCB-transgene and wildtype vaccinia virus (VV) in HEK293A cells. The *MnSOD* expression cassette was introduced into the TK region of vaccinia virus. Pse/L, synthetic early/late promoter. P7.5, vaccinia virus early-late promoter. gpt, mycophenolic acid resistance gene. 5′TK and 3′TK, viral flanking sequences of the TK gene. ITR, inverted terminal repeat. ****, sites of anticipated homologous recombination. (**B,C**) The expression of *MnSOD*. Cells on six-well plates were infected with different viruses at an MOI of 2. After 48 h post-infection, total cellular RNA and protein lysates were extracted to elevate the *MnSOD* expression using real-time PCR and Western blot. (**B**) GAPDH served as an internal control. The data are presented as the mean ± SD of three separate experiments (*** represents *P*<0.001, one-way ANOVA and multiple comparisons). (**C**) Western blot analysis of the apoptosis-related protein caspase in A20 and EL4 cells. β-actin served as a loading control. (**D**) A20 and EL4 cells were infected with MOI: 1 of OVV-MnSOD or OVV, respectively. After an additional 48 h, medium and cells were harvested. The collected supernatant was tested for virus production by standard TCID_50_ assay on 293A cells. Progeny viruses from MOI: 1 of virus were calculated. Data are presented as mean ± SD and the representative of three separate experiments.

