# Supplementary material for: Remodeling of the tumor microenvironment using an engineered oncolytic vaccinia virus improves PD-L1 inhibition outcomes
Source: Biosci Rep. 2021 Jun 10;41(6):BSR20204186. doi: 10.1042/BSR20204186 (PMC8193643; doi:10.1042/BSR20204186)
Supplement: Supplementary Figures S1-S2 [file BSR-2020-4186_supp.pdf]

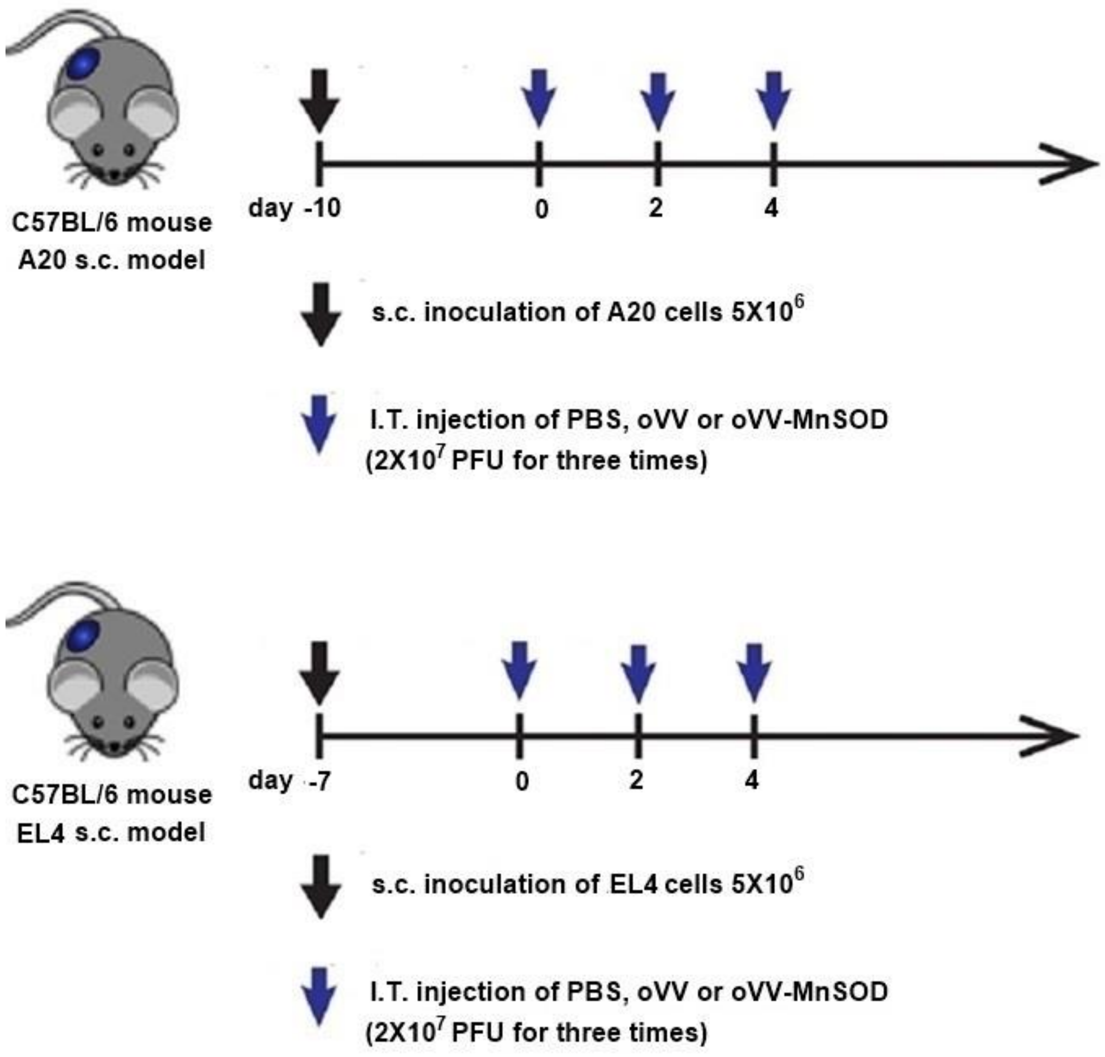

Fig. S1. The mice treatment scheme. The lymphoma subcutaneous (S.C.) model was established by implantation of A20 (5×10^6) EL4 (2×10^6) cells on the right flank of C57BL/6 mice. When the tumor volume reached approximately 100 mm^3 (7 or 10 days post tumor inoculation), the tumor-bearing mice were treated with PBS, 2×10^7 of oVV-Control, or oVV-MnSOD at a 2-day interval for 3 times via intratumoral (I.T.) injection.

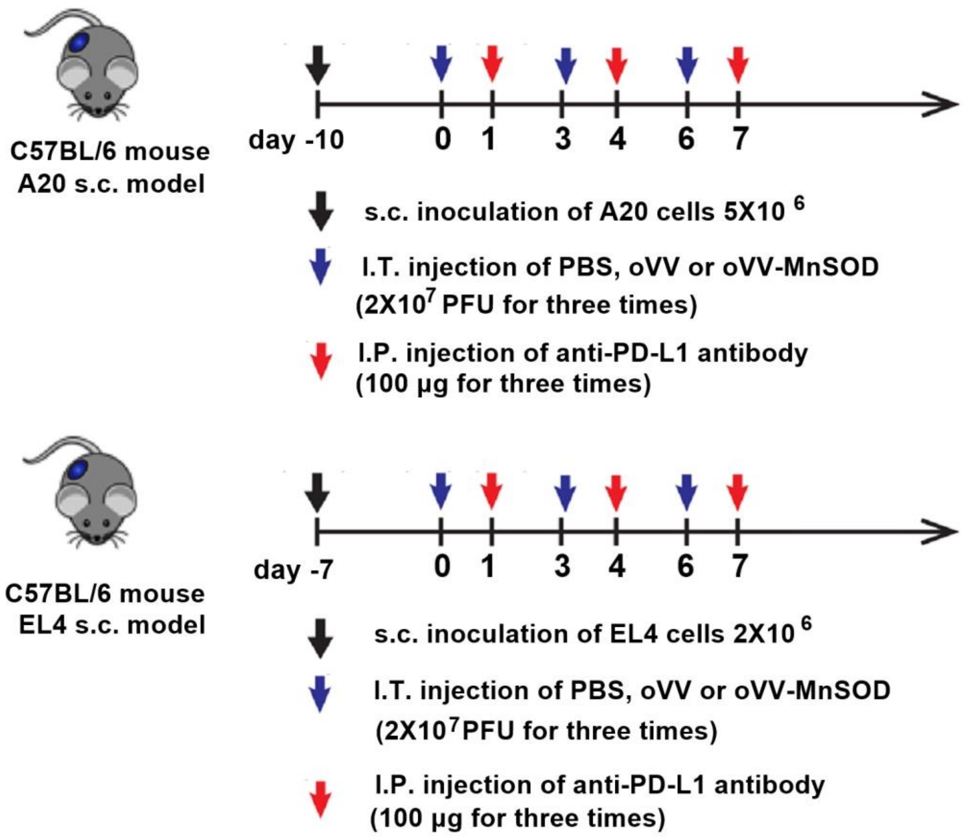

Fig. S2. Schematic diagram of the experiment for combination therapy. Ascites tumor model was established as previously described. The tumor-bearing mice were treated with PBS, 2×10^7 of oVV-Control, or oVV-MnSOD at a 3-day interval for 3 times via intratumoral (I.T.) injection. And 24 hours after each OVs injection, anti-PD-L1 antibody (100 μg per mouse) was injected intraperitoneally (I.P.) into the mouse model for total three times.
